# Geant4-DNA Modeling of Water Radiolysis beyond the Microsecond: An On-Lattice Stochastic Approach

**DOI:** 10.3390/ijms22116023

**Published:** 2021-06-02

**Authors:** Hoang Ngoc Tran, Flore Chappuis, Sébastien Incerti, Francois Bochud, Laurent Desorgher

**Affiliations:** 1 CNRS, CENBG, UMR 5797, Université de Bordeaux, F-33170 Gradignan, France; incerti@cenbg.in2p3.fr; 2 Institute of Radiation Physics (IRA), Lausanne University Hospital and University of Lausanne, CH-1007 Lausanne, Switzerland; Flore.Chappuis@chuv.ch (F.C.); Francois.Bochud@chuv.ch (F.B.); Laurent.Desorgher@chuv.ch (L.D.)

**Keywords:** water radiolysis, RDME, Gillespie, NSM, Geant4-DNA

## Abstract

In this work, we use the next sub-volume method (NSM) to investigate the possibility of using the compartment-based (“on-lattice”) model to simulate water radiolysis. We, first, start with a brief description of the reaction-diffusion master equation (RDME) in a spatially discretized simulation volume (“mesh”), which is divided into sub-volumes (or “voxels”). We then discuss the choice of voxel size and merging technique of a given mesh, along with the evolution of the system using the hierarchical algorithm for the RDME (“hRDME”). Since the compartment-based model cannot describe high concentration species of early radiation-induced spurs, we propose a combination of the particle-based step-by-step (“SBS”) Brownian dynamics model and the compartment-based model (“SBS-RDME model”) for the simulation. We, finally, use the particle-based SBS Brownian dynamics model of Geant4-DNA as a reference to test the model implementation through several benchmarks. We find that the compartment-based model can efficiently simulate the system with a large number of species and for longer timescales, beyond the microsecond, with a reasonable computing time. Our aim in developing this model is to study the production and evolution of reactive oxygen species generated under irradiation with different dose rate conditions, such as in FLASH and conventional radiotherapy.

## 1. Introduction

The energy transfer induced by ionizing radiation in a water medium occurs rapidly (on a scale of femtoseconds (fs)) during the physical stage of water radiolysis and is followed by the formation of radiolytic species. These species are created in a very short time (from femtoseconds (fs) to picoseconds (ps)), mainly through electronic events during the physico-chemical stage [1,2,3,4]. These events—such as thermalization, solvation of sub-excitation electrons, electronic hole migration, and fast electronic recombination—can lead to chemical bond breaks and produce species. After this stage, the chemical stage starts with heterogeneous distributions of radiation-induced spurs along the axis of the tracks from a few picoseconds (ps) to greater than ~100 nanoseconds (ns) in water at room temperature [5]. The spur evolution largely depends on the spatial distribution of these high concentration regions. Methods using Brownian dynamics and Smoluchowski theory have been applied to describe the spur evolution, in which detailed individual trajectories of spherical particles are simulated in chemical reaction-diffusion systems (called "systems" in the following text) [5,6,7,8,9]. In this particle-based representation, only the molecules of interest are explicitly simulated, and the solvent (water) is considered a continuum. Under these conditions, the diffusion of the species occurs until two individual reactants encounter each other (chemical interactions happen when the distance between reactants is less than a pre-defined distance). Over time, the species and their products disperse and are distributed more homogeneously. Their reactions continuously occur until they reach steady-states, where the species concentrations do not change anymore (from 100 microseconds (µs) to several seconds (s)).

Although the detail level is limited when using simplifications (spherical particles, continuum representation), the calculation time remains the main drawback of the particle-based representation when the simulations deal with a large number of species. This makes it difficult to simulate large, absorbed dose radiation systems, where the concentration of produced species may significantly impact the irradiated medium composition. Moreover, the majority of radiation chemistry experiments extend up to the homogeneous regime where radiation-induced species evolve and reach steady-states over long-term scales [5]. Because of its computational cost, the particle-based representation cannot simulate these types of conditions. Instead, compartment-based models can be used because the level of detail is more limited [10].

The compartment-based representation describes species evolution by gathering molecules of the same species as one group placed in a confined volume (or “compartment”). Only molecules in the compartment can react with each other. A basic requirement of compartment-based models is that the distribution of the species is a homogeneous mixture or “well-mixed” in their compartment. Therefore, no information about species positions is described during the evolution. Under this condition, the chemical master equation (CME) [11] is used to describe the time–evolution probability of all the different possible states of the system. In general, the CME is mathematically troublesome. To address this, Gillespie developed a stochastic simulation algorithm (SSA) that equivalently reproduces the CME using a derived Monte Carlo technique [11].

In practice, this “well-mixed” condition is usually not fulfilled since most systems of interest do not begin in homogeneous states. Many of them are first heterogeneously distributed and then tend toward homogeneity as the systems evolve over time, before reaching the fully well-mixed state. For example, in water radiolysis, the transition process between the heterogeneous and homogeneous states may occur continuously from ns to µs or longer, depending on the volume and initial species distribution. Therefore, to be able to apply this well-mixed condition to heterogeneous systems, the compartment-based model proposes dividing the simulation volume into smaller regions (“sub-volume” or “voxels”). In these voxels, the species can be considered as well-mixed (or locally homogeneous). The species can react with each other in the same voxel, and diffusion is modeled by jumps between adjacent voxels. An extension of the CME is the reaction-diffusion master equation (RDME) [12], which is used to account for spatial distributions of species into the states of the system.

In this work, we implement, in Geant4-DNA, the compartment-based (“on-lattice”) model for simulating water radiolysis. In the result and discussion sections, we present several comparisons between the compartment-based model and the SBS method of Geant4-DNA in order to validate our compartment-based model implementation. In the materials and methods section, we detail our implementation of the compartment-based model using the reaction-diffusion master equation (RDME). We also present the next sub-volume method (NSM) [13], the choice of voxels size, and the merging technique using “the hierarchical algorithm for the RDME” (hRDME) [14] approach in our implementation. 

## 2. Results

This section investigates several benchmarks of our new compartment-based model of the water radiolysis, using the particle-based SBS Brownian dynamics model of Geant4-DNA as a reference. In Section 2.1, we verify the modeling of the diffusion by the compartment-based model using the RDME. Then, in Section 2.2, we validate the simulation of a simple reaction-diffusion system. Finally, in Section 2.3, we test the full simulation of water radiolysis under electron beam irradiation using our SBS-RDME model.

### 2.1. Simulation of Diffusion

**Figure 1:**
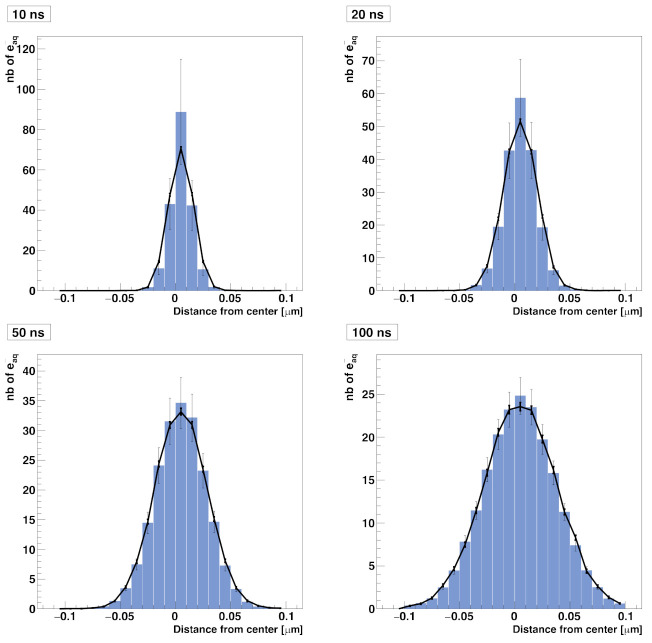
Comparison of spatial (radial) distributions of solvated electrons diffusing in water as computed with the RDME method (blue histogram with unbold error bars) and the SBS method implemented in Geant4-DNA (solid line with bold error bars). The distributions are computed at 10 ns, 20 ns, 50 ns, and 100 ns.

In this test, we simulated the diffusion of solvated electrons (e^−^_aq_) in a 0.2 × 0.2 × 0.2 µm^3^ water cubic volume without chemical reactions taking place. In the compartment-based simulation, the water volume was split into a mesh of 21^3^ voxels with a voxel size of ($h=\frac{0.2}{21}\mu m$) kept constant over time. The simulation started with 200 e^−^_aq_ molecules placed randomly in the central voxel of the mesh. The diffusion coefficient of the solvated electron was set to $D=4.9\times 10^{-9}\;m^{2}\;s^{-1}$, reflecting a temperature of 25 °C. The NSM algorithm (see Section 4.2.1) is only used for diffusion. Figure 1 shows the comparison of spatial distributions of e^−^_aq_ species at time 10 ns, 20 ns, 50 ns, and 100 ns obtained by the compartment-based and the particle-based SBS models. For the particle-based model, since the cubic volume is chosen large enough, we processed four different simulations with one single time step of 10 ns, 20 ns, 50 ns, and 100 ns [6]. We obtained a general good agreement between the results obtained with the compartment-based and the particle-based simulations. Table 1 shows a quantitative comparison of the results by means of Kolmogorov–Smirnov statistical tests. The bad agreement at 10 ns (*p*-value < 0.05) can be explained by the large size of the voxels that resulted in a large time step during this simulation time. At 20 ns, 50 ns, and 100 ns, *p*-values are larger than 0.05, confirming the overall statistical agreement.

### 2.2. Simulation of a Simple Reaction-Diffusion System 

For the second benchmark, we investigated a simplified reaction-diffusion system. We considered the same simulation setup as in Section 2.1 with the exception that the solvated electrons did not only diffuse in water but could also interact with each other (Reaction 9 in Table 3). The resolution of the initial mesh in the compartment-based model should be set small enough to ensure that the molecules are well-mixed in each voxel at the start of the simulation. This condition is fulfilled if the voxel size *h* is smaller than the mean inter-particle distance *d*. The distance *d* can be approximated by the expression ${\left(\frac{V}{N}\right)}^{1/3}$, with *N* the number of primary molecules and V the volume over which they are generated. In our simulation case, *N = 200* and $V=\left(\frac{0.2}{21}\right){\;}^{3}{\;\mu m}^{3},$ and we get *d* = 1.63 nm. We, therefore, selected an initial mesh of 128^3^ voxels, giving a voxel size of *h =* 1.5625 nm, which is smaller than the distance *d*. We then applied the hRDME approach (see Section 4.2.2) to adapt the size of the mesh during the simulation. It started from the initial mesh and changed over time to coarser meshes of 64^3^, 32^3^, 16^3^, 8^3^, 4^3^, 2^2^, and one voxel.

**Figure 2:**
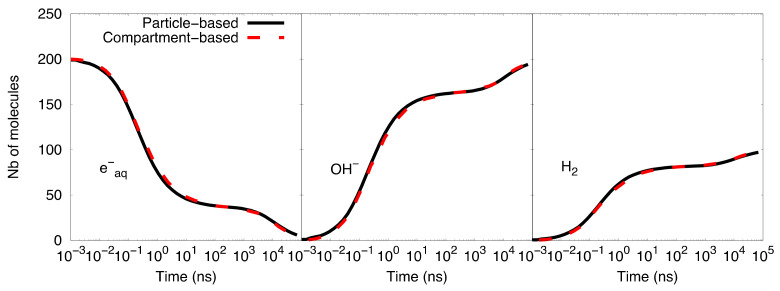
Compartment-based model for the simplified diffusion-reaction system compared with the particle-based SBS simulation.

Figure 2 shows the comparison of the time-dependent yield of species (number of molecules) as computed with the particle-based SBS model and the compartment-based model. A very good agreement was observed between the results obtained with both models. Table 2 shows the speed-up factor of the compartment-based model compared to the particle-based SBS model for different time scales covered by the simulations. The speed-up factor is defined as the ratio between the particle-based SBS and the compartment-based model simulation time. While simulations with end times less than 100 ns show a speed-up factor of one order of magnitude, we observed larger factors for longer timescales. The explanation is that, for timescales from 1 ns to 100 ns, the compartment-based model is performed in fine resolution meshes of 128^3^, 64^3^, 32^3^, and 16^3^ voxels, which makes the compartment-based model less computationally efficient than at longer timescales (from 100 ns to 100 µs) where the system is transferred to coarser meshes. Moreover, at longer timescales, the particle-based SBS is much less efficient than the compartment-based model because it requires using small time-steps when the species reach the volume borders (boundary interactions).

### 2.3. Full Water Radiolysis Simulation

For the third benchmark, we tested our full SBS-RDME model with a water radiolysis simulation. A water box of 0.2 × 0.2 × 0.2 µm was randomly irradiated on one face by a normal incident beam of 1 MeV electrons. The incident electrons were shot until the sum of all energy deposits in the volume led to an absorbed dose of 100 Gy (about 5 keV for the considered water volume). In this simulation, we used the physics list “G4EmDNAPhysics_option2” for the modeling of the physical stage. The microscopic sub-stage (see Figure 6) extended until 1 ns, at which point, we initiated the compartment-based model with a mesh of 32^3^ voxels. We then used the hRDME approach (see Section 4.2.2) to successively transfer species from the finer mesh to coarser meshes of 16^3^, 8^3^, 4^3^, 2^3^, and finally one voxel where the simulation volume is fully homogeneous.

The list of reactions and reaction rates considered in this work are shown in Table 3. It is worth noting that, in this work, these reactions are considered to be diffusion-controlled reactions where the products are created as soon as the reactants encounter each other.

**Figure 3:**
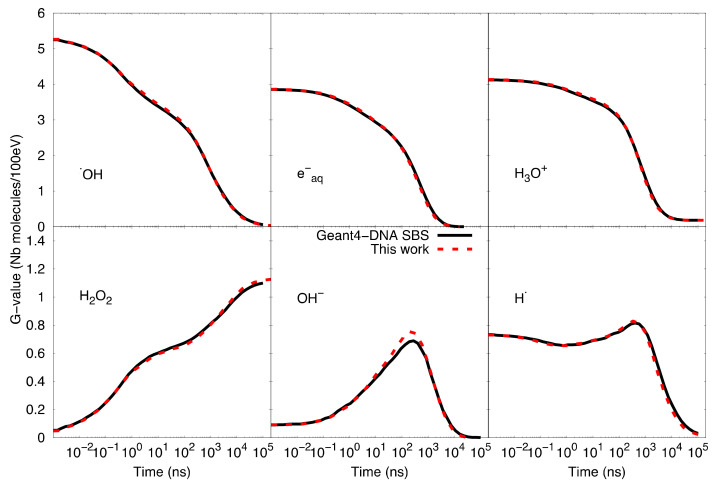
Comparison of time-dependent G-values as computed with the particle-based SBS model and the SBS-RDME model (this work) from 1 ns until 100 µs.

Figure 3 shows the comparison of time-dependent G-values as computed by the particle-based SBS model of Geant4-DNA and our SBS-RDME model. Note that the comparison is considered from 1 ns when the compartment-based model is activated in the SBS-RDME model. A good agreement was observed for the entire time range between both models, thus, validating our SBS-RDME model implementation. Both SBS-RDME and particle-based SBS models showed that the system reached a steady-state at about 100 µs.

Figure 3 also shows a slight imbalance of neutrality of about 0.18 molecules per 100 eV observed in the G-value of H_3_O^+^. The explanation is that during the physical stage, some secondary electrons left the simulated volume as a result of ionizations that took place near the volume borders. These escaping electrons would not produce solvated electrons in the considered volume, while the corresponding ionized water molecules would dissociate in the volume and, therefore, create the slight imbalance of neutrality.

## 3. Discussion 

A challenging aspect of our method is the choice of the time *t*_1_ when the microscopic sub-stage ends, and the mesoscopic sub-stage starts (see Section 4). The distribution of energy deposits by ionizing particles produces regions of microscopic spurs along the tracks. Since spur sizes are comparable with their reaction radius within a few ns after exposure up to the time *t*_1_, the microscopic sub-stage cannot be described by using the “well-mixed” model. After this period, local homogeneity eventually appears, and the compartment-based model can be applied. A small *t*_1_ could lead to a small initial voxel size, which may cause unphysical results, while a long *t*_1_ could make the simulation less efficient, especially for systems with a large number of species. Moreover, *t*_1_should also depend on the initial species distribution in which the linear energy transfer (LET) of the irradiation source is involved. However, the method to determine *t*_1_ for different LETs is beyond the scope of this work.

In the compartment-based model, the splitting of the simulation volume into smaller volumes helps to improve the computation accuracy. Indeed, in a simple system of unimolecular reactions, Isaacson [16] showed that a deterministic partial differential equation model can be exactly reproduced from the RDME when the voxel size *h* tends to 0. However, the voxel size should not be too small. The first reason is that the voxel size must be much larger than the reaction radii to maintain the physical validity of the RDME. Since reactive events involve reactants in the same voxel, a voxel size *h* comparable to the reaction radius may lead to missing reactions. This value should not be less than a critical size of about πσ, where σ is the reaction radius [17]. The second reason concerns the computational efficiency of the model. When the voxel size is too small, most events are diffusions with a small step length (see Equation (4)). These repetitive diffusion events slow down the simulations. Therefore, the key question is how to adapt the appropriate voxel size *h* to the evolution of the system. The answer depends on each particular case. In water radiolysis, the evolution of the radiolytic species is complex. The simulation configuration such as the beam shape, the irradiated medium, temperature, etc., may significantly change the output of the system. However, for a simplified system, for example, a system in which radiation is instantaneously absorbed and the evolution of produced species starts simultaneously, we observed that the voxel size *h* could be smaller at the beginning when the species were close to each other and then became increasingly coarser over time when the species distributed more homogeneously by diffusion. The coarser meshes helped in reaching longer time steps and, therefore, reduced the computational time.

To change the mesh size during the simulations, we needed to know the spatial distribution of the species over time. The first step of the NSM algorithm (see Section 4.2.1) implies that the compartment-based representation should start from the particle-based representation by filling particle position information at the end of the microscopic sub-stage. After that, no more information on species positions is kept, and we cannot use particle position to change the voxel size. A good strategy would be to track particle position with compartment-based representation in a hybrid method [18]. However, this may increase the computational cost when dealing with a large number of species. The solution used in this work was to merge eight adjacent octant voxels into one and, thus, obtain larger voxels (“hierarchical algorithm for the RDME—hRDME”) [14]. Therefore, all species concentrations were initially distributed in a fine mesh and then transferred to coarser meshes over time until we reached the coarsest mesh of one single voxel. Thus, the transfer time $\Delta t_{n}$ should be large enough for the random migration by diffusion of species between the constitutive voxels to reach a “well-mixed” state in their coarser mesh.

**Figure 4:**
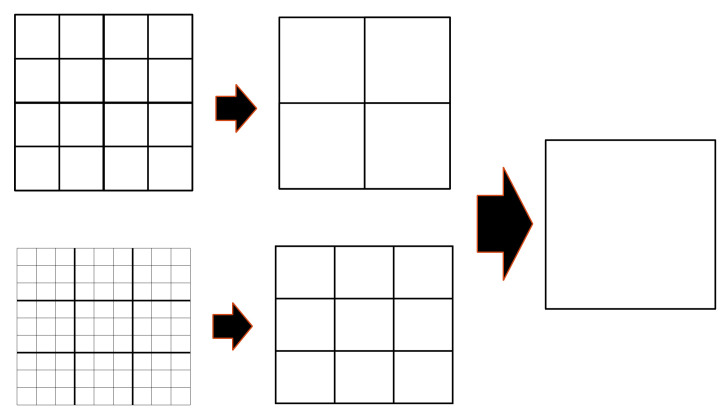
The hRDME approach, here represented in 2D. The mesh of 4^3^ voxels (**top**) is moved to 2^3^, and then to one voxel. The mesh of 9^3^ voxels (**bottom**) is moved to 3^3^ and then to one voxel.

**Figure 5:**
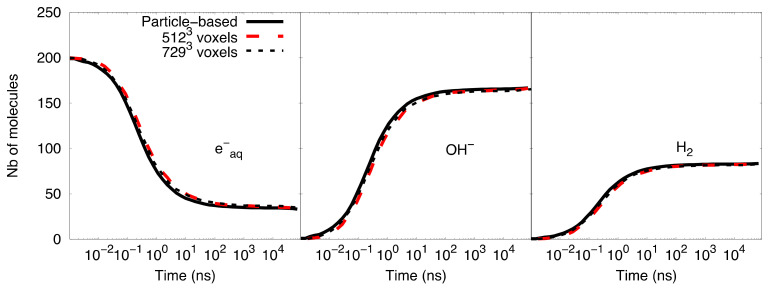
Comparison of species yield between the compartment-based model (the initial mesh of 729^3^ voxels and the initial mesh of 512^3^ voxels) and the particle-based SBS model.

An example of the eight-octant merging operation used in the hRDME method is shown in 2D at the top of Figure 4, where the mesh of 4^3^ voxels moves to 2^3^ voxels and then to one single voxel. This algorithm can also be extended to the twenty-seven-voxels merging operation, where 27 voxels were merged into one larger voxel. For example, an initial mesh of 9^3^ voxels was moved to 3^3^ and then one voxel (see bottom of Figure 4). In this case, the changes of size between the finer and coarser meshes were larger than with the eight-octant merging operation, while the well-mixed requirement was still maintained since the transfer-time $\Delta t_{n},$ for the random migration is longer in Equation (5). These larger changes of size not only speed up the simulation but also relax the choice of initial meshes, which are limited by the physical validity and the hierarchical requirement. This choice is particularly important for systems having a large geometrical volume because it helps to split the volume into a large number of voxels to start with a sufficiently fine mesh. For illustrative purposes, we considered the simple reaction-diffusion system tested in Section 2.2, for which we extended the simulation volume to 1 × 1 × 1 µm^3^ dimension. In order to keep a good start resolution close to the mean inter-particle distance, the initial mesh in the eight-octant merging operation was constrained by a fixed number of voxels equal to 512^3^ or 1024^3^. While an initial mesh of 1024^3^ voxels involved a resolution of 0.976 nm, which is shorter than the critical voxel size (πσ = 1.13 nm), an initial mesh of 512^3^ voxels gave a resolution of 1.9531 nm, which is larger than the mean inter-particle distance (1.63 nm) (see Section 2.2). In this case, the twenty-seven-voxels merging operation is a better choice as an initial mesh of 729^3^ voxels and provides a resolution of 1.37 nm. Figure 5 shows the comparison of the time-dependent yield of species (number of molecules) as computed with the particle-based SBS model and the compartment-based model starting with 729^3^ voxels and 512^3^ voxels. A better agreement of the species evolution was found between the compartment-based model, starting with an initial mesh of 729^3^ voxels, and the particle-based SBS simulation, while the initial mesh of 512^3^ voxels shows a slight deviation. This comparison illustrates the fact that the choice of initial meshes is not straightforward in the compartment-based model, as it depends on the type of simulated system.

Finally, another constraint of the compartment-based model presented in this work is the cubic volume of the simulation. This volume shape makes it possible to create uniform Cartesian meshes, which are particularly simple and efficient to handle in compartment-based simulations. A technical challenge of the compartment-based model would be to extend it to treat all forms of volumes with, for example, curved boundaries.

## 4. Materials and Methods

**Figure 6:**
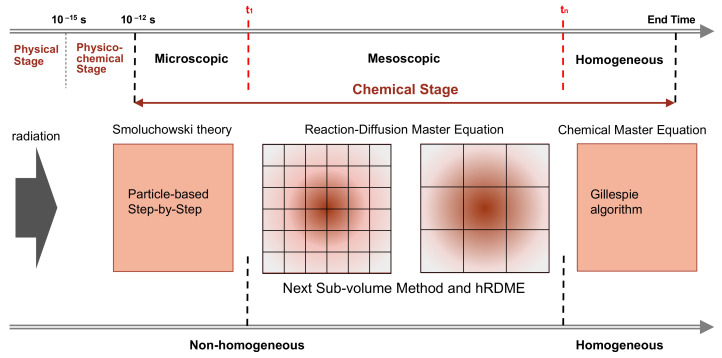
The simulation scheme of the combination of the particle-based SBS model with the compartment-based model that uses the RDME (SBS-RDME model).

The main characteristic of our model is the combination of the SBS Brownian dynamics model already available in Geant4-DNA with the compartment-based model using the RDME (so-called “SBS-RDME model”) in water radiolysis simulations. Figure 6 illustrates the simulation scheme of this combination.

We began by using Geant4-DNA [19,20,21,22,23,24,25] to model the physical and physico-chemical stage up to 1 ps. Then, the chemical stage was divided into three sub-stages: the microscopic, mesoscopic, and homogeneous sub-stages. The microscopic sub-stage started from the formation of species at the beginning of the chemical stage. As the spatial distribution of the early species (e^−^_aq_, H^•^, ^•^OH, H_3_O^+^,…) was concentrated in very small volumes around the tracks, their evolutions largely depended on the species position. Therefore, at this sub-stage, we used the particle-based SBS method of Geant4-DNA to simulate the detailed trajectories of the individual species. Once the simulation reached a time *t_1_*, we stopped the particle-based SBS simulation and initiated a uniform 3D Cartesian mesh for the compartment-based model. This was the beginning of the mesoscopic sub-stage. The initial mesh resolution should be small and selected according to the spatial distribution of species at the end of the microscopic sub-stage. We then used the hRDME approach (see details in Section 4.2.2) to adapt the size of the voxels during the evolution of the system. The system used increasingly coarser meshes over time, to finally merge into one single voxel that covered the full simulation volume at time *t_n_*, when the homogeneous sub-stage started. During the homogeneous sub-stage, we used the CME stochastic process to sample only reactive events.

### 4.1. The Microscopic Chemical Stage

During the microscopic sub-stage, the species diffused, and the reactions happened as soon as the distance between two species was shorter than their reaction radius [6]. The diffusion and reaction processes were simulated using the particle-based step-by-step (SBS) method, which is available in Geant4-DNA [6]. Briefly, this method proposes a time-step model that allows the choice of time steps, during which the reaction cannot occur with at least 95% (by default) confidence (named “dynamic time step”). Therefore, this model requires a minimum time step to avoid the repetition of many small time steps. For this work, we set the minimum time step at 1 ps. Details of the step-by-step (SBS) method can be found elsewhere [6,8].

### 4.2. The Mesoscopic Chemical Stage

**Figure 7:**
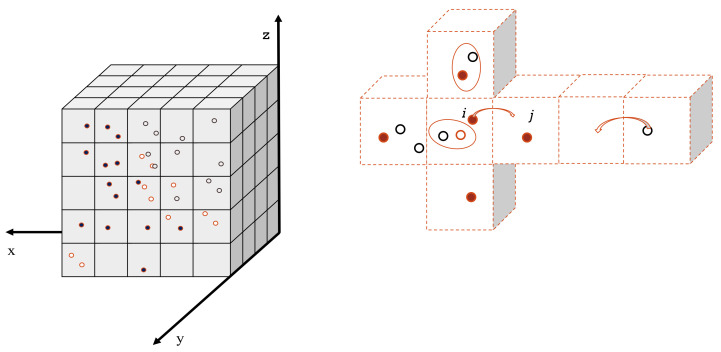
(**Left**)/Voxelization of the considered volume of simulation into smaller sub-volumes. Species are represented by different types of circles. (**Right**)/The reactions and diffusion are triggered independently in a random process.

During the mesoscopic stage, we employed the compartment-based model, using the reaction-diffusion master equation (RDME) to simulate the heterogeneous chemical phase. The principal characteristic of this model is that it divides the simulation space into voxels, inside which the medium is considered homogeneous (Figure 7—left). Reactions between molecules take place within the voxels, while species can move from one voxel to an adjacent one by diffusion (Figure 7—right). We defined the propensity function (Equation (1)) of the RDME so that its product with a given infinitesimal time step provides the probability that the given event (reaction or diffusion) occurs in this time step. For a given voxel *i*, it is given by the following equation.

(1) $$a_{i}=\overset{R}{\underset{r=0}{\sum }}a_{i}^{r}+\overset{M}{\underset{\begin{array}{c} j=1\\ j\neq i \end{array}}{\sum }}\overset{L}{\underset{k=1}{\sum }}d_{i,j}^{k}$$

The first term $a_{i}^{r}$ is the propensity function of reaction *r* among R possible bimolecular reactions (or “second-order” reactions) that is written as:

(2) $$a_{i}^{r}=\left\{\begin{array}{c} \;\;\;S_{m}*S_{l}*\frac{k_{r}}{V_{i}},\;\;if\;m\;\neq l\\ \frac{1}{2}S_{m}*(S_{l}-1)*\frac{k_{r}}{V_{i}},\;\;if\;m=l\; \end{array}\right.$$

where $S_{m}$, $S_{l}$ are the number of the reactants m and l in the voxel *i*, respectively, $k_{r}$ is the reaction rate, and $V_{i}$ is the volume of the voxel *i*.

The second term $d_{i,j}^{k}$ in Equation (1) is the propensity function of the diffusion when a molecule of species *k* among *L* existing species is transferred from voxel i to voxel j among the *M* possible adjacent voxels. It is written as:

(3) $$d_{i,j}^{k}=\lambda *S_{k}$$

where $S_{k}$ is the number of the chemical species k in the voxel *i* and λ is the diffusion rate. The diffusion rate is given by:

(4) $$\lambda =\frac{D}{h^{2}}$$

where *D* is the diffusion constant ([length]^2^[s]^−1^) and *h* is the cubic voxel size ([length]).

The diffusion is also called the “diffusive transfer reaction” [12]. Indeed, diffusion can be considered a sequence of a degradation reaction in voxel i and a production reaction in voxel j. Both reactions are first-order reactions that are handled by the Gillespie algorithm, as are second-order reactions between species (see Section 4.2.1).

#### 4.2.1. Sampling of Next Reaction/Diffusion Event

To establish the list of reactive and diffusive events over all voxels, we used the next sub-volume method (NSM), introduced by Elf and Ehrenberg [13]. Since these events are scheduled independently in each voxel state, the NSM has an event-driven nature where, at every time step, an event occurs. This event-driven nature enables the NSM to efficiently organize events in a queue and process them one by one. The sampling of these events in each voxel is computed by using the direct method of the Gillespie algorithm [11,12]. In order to choose the voxel in which the next event occurs, the NSM applies the next reaction method [26].

The numerical implementation of the NSM is summarized here [13]:

(1) Computation of the species concentrations over N voxels of the initial mesh at time *t*_1_.
(2) Calculation of the propensity functions $a_{i}$ for all voxels (see Equation (1)).
(3) Sampling of the time when the next event occurs for all the voxels,
  $$\tau_{i}=\frac{-\ln\left(\xi \right)}{a_{i}}$$
  where ξ is a uniform random number in [0,1].
(4) Sampling which types of reactions or diffusion will take place at a time $\tau_{i}$ according to their relative contribution ($a_{i}^{r}$, $d_{i,j}^{k}$) in the propensity function $a_{i}$.
(5) Sorting these events according to their occurrence times $\tau_{i}$.
(6) Processing the first event in the queue and changing the concentrations in the voxels involved in the event.
  - If the event is a reaction, we eliminate reactants and create products.
  - If the event is a diffusion, we remove the particle in the voxel where it was located and add the particle in the voxel where it goes.
(7) Sampling of $\tau_{i}$ and the type of events in the updated voxels according to their new concentrations following points 3 and 4 and sorting the new list of events, as in point 5.
(8) The queue of events is processed successively following points 3 to 7 until no more events are present, or the times $\tau_{i}$ are longer than the end time of the simulation.

#### 4.2.2. Adaptation of Voxel Sizes during the Evolution Time

To adapt the voxel size, we applied the hierarchical algorithm for the RDME (hRDME) [14]. This method consists of merging the eight octant adjacent voxels (so-called “eight-octant merging operation”) into a single larger voxel. For example, an initial mesh of 32^3^ voxels is gradually merged into a mesh of 16^3^, 8^3^, 4^3^, 2^3^, and finally into one voxel. All species of finer voxels are moved to a larger voxel of a coarser mesh after each “transfer time”$\;\Delta t_{n}$. In our implementation, we took the transfer time as proposed in the study of Hellander and Hellander [14]. It is written:

(5) $$\Delta t_{n}=20\frac{h_{n}{}^{2}\;}{6D}$$

where $h_{n}$ is the voxel size corresponding to the transfer-time $\Delta t_{n}$, and D is the lowest diffusion constant among all species.

### 4.3. The Homogeneous Chemical Stage

The hRDME algorithm changes the voxel size until there is only one voxel covering the whole simulation volume. The species in the volume are considered to be well-mixed, and the Gillespie algorithm using CME is applied to sample only reactive events. The homogenous sub-stage ends when the system enters the steady-state.

### 4.4. Bounded Volume

In our simulation, we considered that molecules diffuse and react in a bounded volume (that is, limited by geometrical boundaries), which is also the irradiated water box volume of the physical stage. For the first time, we implemented the reflective boundary conditions [27] in the SBS method of Geant4-DNA. These conditions enabled us to confine the chemical molecules in the considered volume by bouncing them off the walls of the volume. We also applied the bouncing off the volume border for the mesoscopic and homogeneous sub-stages.

### 4.5. G-Values

We calculated the yield of a chemical species induced by water radiolysis in terms of G-value. This represents the mean number of molecules of the species produced per 100 eV of energy imparted.

## 5. Conclusions

In this work, we implemented, in Geant4-DNA, the compartment-based model combined with the SBS Brownian dynamics model already available in Geant4-DNA. We showed that the compartment-based model can reproduce the same species yield obtained by the particle-based SBS of Geant4-DNA but with 100 to 1000 times less computing time. Moreover, our new model can also extend the simulation timescale beyond the microsecond when the system has reached a steady-state. These advantages will allow us to study the production and evolution of reactive oxygen species generated under irradiation with different dose rate conditions, such as in FLASH and conventional radiotherapy.

In this work, we only considered a radiation-induced species system in a cubic water volume. The approach could be applied for more complicated systems, such as complex geometries including biological materials. This may be technically more difficult since we need to handle the two different representations (compartment-based and particle-based) in one simulation. We plan to work on such an extension in the future.

## Figures and Tables

**Table 1:** Kolmogorov–Smirnov statistical tests for comparison of spatial (radial) distributions obtained with the compartment-based and particle-based simulations.

Distribution at Time	Kolmogorov-Smirnov Test
10 ns	D = 0.428571 *p* = 0.042256
20 ns	D = 0.333333 *p* = 0.193767
50 ns	D = 0.095238 *p* = 0.999981
100 ns	D = 0.095238 *p* = 0.999981

**Table 2:** Speed-up factor of the compartment-based model compared to the particle-based SBS model for different simulation end times from 1 ns to 100 µs.

**End time**	1 ns	10 ns	100 ns	1 µs	10 µs	100 µs
**Speedup factor**	13	15.7	16.5	2.5 × 10^2^	2.3 × 10^3^	2.3 × 10^4^

**Table 3:** The reactions and reaction rates [15] used in this work that apply at ambient temperature (25 °C).

	Reaction	Reaction Rate (10^10^ M^−1^s^−1^)
1	H^•^ + e^−^_aq_ + H_2_O → OH^−^ + H_2_	2.5
2	H^•^ + ^•^OH → H_2_O	1.55
3	H^•^ + H^•^ → H_2_	0.503
4	H_2_O_2_ + e^−^_aq_ → OH^−^ + ^•^OH	1.1
5	H_3_O^+^ + e^−^_aq_ → H^•^ + H_2_O	2.11
6	H_3_O^+^ + OH^−^ → 2H_2_O	11.3
7	^•^OH + e^−^_aq_ → OH^−^	2.95
8	^•^OH + ^•^OH → H_2_O_2_	0.55
9	e^−^_aq_ + e^−^_aq_ + 2H_2_O → 2OH^−^ + H_2_	0.636

## Data Availability

Not applicable.
